# Opportunities and Challenges in Studies of Host-Pathogen Interactions and Management of *Verticillium dahliae* in Tomatoes

**DOI:** 10.3390/plants9111622

**Published:** 2020-11-22

**Authors:** Bhupendra Acharya, Thomas W. Ingram, YeonYee Oh, Tika B. Adhikari, Ralph A. Dean, Frank J. Louws

**Affiliations:** 1 Department of Entomology and Plant Pathology, North Carolina State University, Raleigh, NC 27695, USA; bachary2@ncsu.edu (B.A.); twingra2@ncsu.edu (T.W.I.); yoh2@ncsu.edu (Y.Y.O.); radean2@ncsu.edu (R.A.D.); 2 Department of Horticultural Science, North Carolina State University, Raleigh, NC 27695, USA

**Keywords:** tomato, *Solanum Lycopersicon* L., *Verticillium dahliae*, plant-pathogen interactions, disease resistance, integrated disease management

## Abstract

Tomatoes (*Solanum lycopersicum* L.) are a valuable horticultural crop that are grown and consumed worldwide. Optimal production is hindered by several factors, among which *Verticillium dahliae*, the cause of Verticillium wilt, is considered a major biological constraint in temperate production regions. *V. dahliae* is difficult to mitigate because it is a vascular pathogen, has a broad host range and worldwide distribution, and can persist in soil for years. Understanding pathogen virulence and genetic diversity, host resistance, and plant-pathogen interactions could ultimately inform the development of integrated strategies to manage the disease. In recent years, considerable research has focused on providing new insights into these processes, as well as the development and integration of environment-friendly management approaches. Here, we discuss the current knowledge on the race and population structure of *V. dahliae,* including pathogenicity factors, host genes, proteins, enzymes involved in defense, and the emergent management strategies and future research directions for managing Verticillium wilt in tomatoes.

## 1. Introduction

### 1.1. Economic Importance of Tomatoes

**Figure 1:**
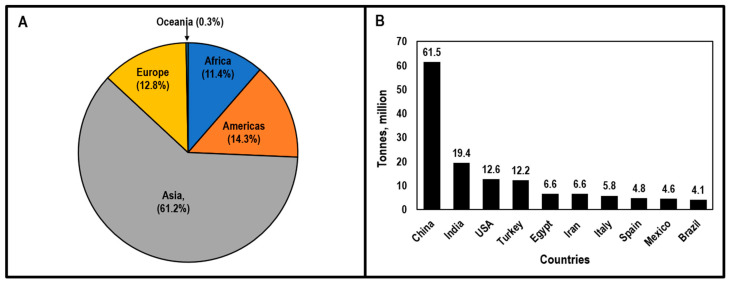
Tomato production by region (**A**) and top 10 tomato-producing countries in the world in 2018 (**B**). Adapted and modified from [1]

Tomatoes (*Solanum lycopersicum* L.) are an important fruiting vegetable grown around the world, with Asia being responsible for more than 50% of the total production (Figure 1A). In 2018, approximately 182.3 million tons of tomatoes were harvested from 4.8 million ha of land worldwide and according to Food and Agriculture Organization Statistics (FAOSTAT) [1] China (61.5 Mt), India (19.4 Mt), and the United States (12.6 Mt) were the top three tomato-producing countries (Figure 1B). Depending on the part of the world where tomatoes are grown, major constraints in tomato production include a lack of quality seeds, labor, and knowledge of optimum agronomic practices; the high cost of agricultural inputs and price fluctuations; weather constraints; and the serious problem of insect pests and diseases [2,3,4,5,6]. Among tomato diseases, Verticillium wilt is a major biological constraint of tomato production.

### 1.2. Life Cycle of Verticillium dahliae and Symptoms in Tomatoes

**Figure 2:**
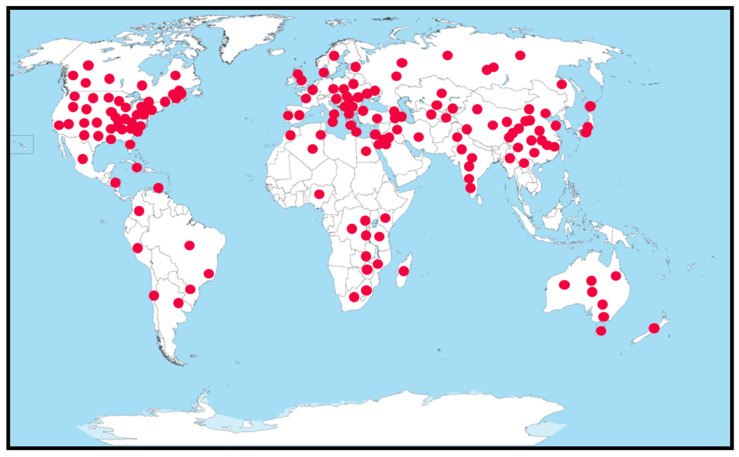
Worldwide distribution of *Verticillium dahliae*. Circles represent the locations (states and provinces) where *V. dahliae* has been reported. Adapted from [16].

*Verticillium dahliae* is a hemibiotrophic fungal pathogen [7] with a worldwide distribution (Figure 2) that causes Verticillium wilt in tomatoes and many other crops [8]. The disease cycle starts with microsclerotia (MS), a resting structure in soil or crop debris that is capable of surviving without a plant host for more than a decade [8,9]. Disease severity is linked to MS density (MS/g soil), which can be quantified using several techniques [10,11,12,13,14], but the methods have not been refined for routine use in commercial labs to reliably guide disease management decisions. Inoculum density as low as 0.1 microsclerotia (MS)/g of soil is sufficient to infect tomato plants, but even levels of 9 MS/g in the soil do not always yield visible symptoms [15]. Moreover, the level of infection from the same amount of inoculum depends on environmental conditions. This complicates the determination of economic thresholds for this pathogen as a basis for the application of integrated disease management approaches.

The microsclerotia germinate in the presence of root exudates [17], and mycelium infect roots through root tips or sites of lateral root formation [18,19]. The typical symptoms of *V. dahliae* in susceptible tomato cultivars start on the lower leaves, with chlorosis and V-shaped necrotic lesions at the edges of the leaves with yellow halos that expand to cause browning or purpling of veins and death of the leaves [20,21]. The pathogen spreads acropetally through the vascular tissue of the plant, and brown discoloration is visible when incised [20], producing conidia that continue the cycle of germination, infection, and colonization, resulting in the wilting of branches and/or the entire plant [19]. Prolific conidiation has been correlated with the aggressiveness of the strains [18]. Although the appearance of wilting symptoms in a susceptible tomato cultivar depends on the virulence of the pathogen and other environmental factors, the accumulation of drought-stress related proteins has been correlated with the beginning of wilting symptoms at 21 days post-infection (dpi) [22].

The broad host range, which includes annuals, perennials, and woody species, comprises more than 200 plant species [8,23], and its ability to persist in soil for a long period makes *V. dahliae* an important and widely studied pathogen. Several studies have focused on *V. dahliae* biology, host-pathogen interactions, and recent approaches to managing the disease.

## 2. Current Knowledge on *Verticillium dahliae* and Host Interactions 

### 2.1. Race Structure in Verticillium dahliae

#### 2.1.1. *Verticillium dahliae* Race 1 Infecting Tomatoes

A single dominant locus, *Ve,* conferring resistance to race 1 isolates of *V. dahliae* and *V. albo-atrum*, was identified in 1951 [24]. Virulent isolates were discovered within a few years after the deployment of this resistance [25,26,27,28,29]. In 1984, Bender and Shoemaker surveyed 96 *V. dahliae* isolates; 89 were designated as race 1, and seven were non-race 1, using differential tomato lines with and without the *Ve* gene [26]. The gene responsible for race 1 resistance in tomatoes, and other hosts, designated as the *Ve1* gene, has been described using a combination of whole-genome comparison and gene expression analyses [30]. The *Ve1* gene codes for a cell surface-like receptor which recognizes the Ave1 effector of the pathogen during the infection process [30,31]. Homologs to *Ave1* exist in *Colletotrichum*, *Fusarium*, and *Cercospora* [30]. The *Ve1* gene can also activate the immune response against *Xanthomonas axonopodis,* which causes citrus canker [30]. Interestingly, *Ave1* can also induce defensive gene expression in the absence of the *Ve1* gene [32], which suggests other defense responses independent of Ve1 may also be operating.

#### 2.1.2. *Verticillium dahliae* Race 2 Infecting Tomatoes

Isolates pathogenic to race 1 resistant plant are present globally [26,27,33,34,35,36,37]. These isolates have been classified as race 2 in the past; however, they are better classified as non-race 1 and comprise race mixtures. In 2017, the cultivars ‘Aibou’ and ‘Ganbarune-Karis’ were shown to be resistant to some non-race 1 strains of *V. dahliae* [37]. ‘Aibou’ and ‘Ganbarune-Karis’ are F1 hybrids. F2 progeny of these lines segregated into a 3:1 resistant to susceptible ratio, suggesting the presence of a single dominant resistance gene. Isolates that were non-pathogenic to ‘Aibou’ and ‘Ganbarune-Karis’ were termed race 2, while isolates pathogenic to these cultivars were termed race 3 [37]. Further research has shown that knocking out the race 1 effector in isolate Vdp4 (a race 1 strain of *V. dahliae*) was pathogenic to ‘Aibou’, which contains both race 1 and race 2 resistance [38]. Kano and Usami [37,38] also showed that one isolate (Vdp4) was non-pathogenic to race 1 resistant plant but pathogenic to tomatoes containing just the race 2 resistance gene. Furthermore, they demonstrated that some race 1 isolates were non-pathogenic to a cultivar that was susceptible to race 1 but resistant to race 2, suggesting that some race 1 isolates also contain the race 2 effector. Ingram et al. (unpublished data) showed that race 2 and race 3 isolates are present in the USA in isolates from tomatoes in North Carolina and California (Table S1). An analysis of the genomes of Japanese and USA races 1, 2, and 3 isolates showed that there are three candidate secreted effectors that may be responsible for the race 2 phenotype [39], demonstrating that one of these secreted effectors is responsible for the race 2 phenotype. The race 2 secreted effector was introduced into race 3 strains, which then exhibited the race 2 phenotype when inoculated onto tomato lines containing the V2 locus [39]. The host resistance gene responsible for the race 2 resistance phenotype is currently unknown.

### 2.2. Influence of Genetics on Verticillium dahliae Pathogenicity

#### 2.2.1. Defoliating (DF) vs. Non-Defoliating (NDF) Strains on Tomatoes

In cotton, there are two radically different pathotypes of *V. dahliae*; the non-defoliating (NDF) and defoliating (DF) strains [40,41]. DF strains of *V. dahliae* cause a massive amount of damage to cotton plants [42]. While primers exist to differentiate these pathotypes, some strains that are polymerase chain reaction (PCR) positive (such as VdLs17) for DF do not have the DF phenotype [42]. However, the DF strains are not pathogenic to tomatoes [43]. The DF phenotype is caused by the presence of the *VdDf5* and *VdDf6* genes, which are contained in a lineage-specific region that was horizontally transferred from *Fusarium oxysporum* f. sp. *vasinfectum* to *V. dahliae* [43].

#### 2.2.2. Vegetative Compatibility of *Verticillium* *dahliae* Isolates

*Verticillium dahliae* is an asexually reproducing haploid ascomycete fungus in the class sordariomycetes, which is a class that contains many plant pathogenic fungi [7,44,45]. Despite the presence of two mating types (MAT1-1 and MAT1-2), there is very little evidence of recombination [16,46]. However, there are vegetative compatibility groups (VCGs) which may allow for some parasexual exchange of genetic material [47,48]. Because of the highly clonal nature of *V. dahliae*, VCGs were used in the past to differentiate isolates, although it is unclear whether there are any direct links to pathogenicity [47,49]. In 2017, isolates from strawberries containing the race 1 effector *Ave1* were grouped into two different VCGs, and two race 1 VCG groups were also phylogenetically different from each other [50]. Overall, there is very little information to suggest *V. dahliae* isolates exchange any genetic material at all, and claims of VCGs affecting pathogenicity should be examined on a case-by-case basis.

#### 2.2.3. Chromosomal Rearrangement and its Influence on Pathogenicity in *Verticillium* *dahliae*

To date, considerable variation in the pathogenicity of *V. dahliae* isolates has been attributed to either chromosomal rearrangement or horizontal gene transfer events from other organisms [51,52,53]. The absence of *Ave1* in non-race 1 strains of *V. dahliae* is due to the absence of a large region on the chromosome where the effector is located [52]. *Ave1* is homologous to plant natriuretic peptides (PNPs), which are secreted peptides that regulate abiotic stress in plants [52,53]. Similarly, the absence of the Av2 locus in race 3 is the result of a large deletion on chromosome 5 (JR2 reference genome) [39]. A large number of insertions and deletions in *V. dahliae* have led to the hypothesis of a two-speed genome, where vital genes are kept in specific regions, while pathogenicity-related genes are located on more flexible regions with transposable elements (TEs) [54,55,56]. Genomic plasticity appears to be a major force driving the host-pathogen evolution of *V. dahliae* and other fungal pathogens [55,56,57].

#### 2.2.4. Phylogenetic Analysis of *Verticillium* *dahliae* Isolates

Microsatellite data and whole-genome sequencing have been effective ways to differentiate *V. dahliae* populations [7,44,46]. The largest most comprehensive phylogenetic analysis of *V. dahliae* isolates to date was conducted by Short et al. [58] on 1100 *V. dahliae* isolates from a wide range of hosts and continents, using microsatellite genotyping. The study indicated that there are seven distinct clusters, and isolates from tomatoes were present in clusters 1, 2, and 7 [58], which included the sequenced tomato *V. dahliae* isolates, Le1811 and Le1087, and the lettuce isolate VdLs17 (alternatively labeled as PD322). In 2020, Ingram et al. (unpublished data) circumscribed at least two supergroups and four sub-groups of *V. dahliae* isolates infecting tomatoes. Whole-genome analysis has yielded a great deal of information into this pathogen evolution [43,44,58].

Phylogenetic analysis was complicated by the existence of a diploid hybrid, *V*. *longisporum*, a long-spore hybrid between *V. dahliae* and a cryptic *Verticillium* species [59,60,61]. The evolutionary history of *V. longisporum* suggested three separate hybridization events have occurred between two different *Verticillium* spp. [60]. Population genetic analysis reveals that *V. dahliae* and the cryptic *Verticillium* species form three distinct hybrid lineages (termed A1/D1, A1/D2, A1/D3), and genome hybridization among a limited number of species indicates evidence of divergent evolution [60]. Furthermore, comparative virulence analysis among isolates of the three *V. longisporum* lineages indicated that lineage A1/D1 isolates were the most virulent on oilseed rape, while lineage A1/D2 isolates were the most virulent on cabbage and horseradish [62]. *Verticillium longisporum* isolates were virulent on several non-Brassicaceae hosts such as eggplants, tomatoes, lettuce, and watermelon, and these results suggested that *V. longisporum* has a wider host range and is more virulent than *V. dahliae* [62].

### 2.3. Molecular Insights into Verticiliium dahliae Pathogenicity

Molecular genetics and other ‘omics’ technologies have been widely used to uncover the molecular basis of pathogenicity in *V. dahliae* in recent years. *V. dahliae* is phylogenetically closely related to other foliar and soilborne pathogens. Consequently, homology-based approaches have been exploited in several instances to identify and characterize genes and pathways known to be involved in the development and pathogenicity in other pathogens. For example, hydrophobin, a small secreted hydrophobic protein, is known to be essential for fungal development and pathogenicity [63]. The study of a *V. dahliae* homolog (VDH1) showed that hydrophobin is essential for microsclerotia formation but is not required for host colonization and pathogenicity [64].

Several proteins involved in core fungal processes, such as cell wall modification, play crucial roles in cell wall integrity and pathogenicity. Mannoproteins, which are rich in fungal cell walls (in the range of 30–50% in yeast cell walls), are connected to the cell wall via either non-covalent connections or covalent linkages to β-1,6-glucans. Alpha-1,6-mannosyltransferase (OCH1) is required to produce yeast mannoproteins [65]. In *V. dahliae*, an OCH1 homolog is required for both microsclerotia formation and pathogenicity [66].

Genes for energy metabolism have been characterized in *V. dahliae,* and in some instances play a role in pathogenicity. The enzyme alpha-oxoglutarate dehydrogenase (OGDH) catalyzes the oxidative decarboxylation of alpha-ketoglutarate to succinyl-CoA in the tricarboxylic acid (TCA) cycle. VdOGDH in *V. dahliae* is not only involved in energy metabolism but also affects the expression of melanin biosynthesis and is required for full virulence [67].

#### 2.3.1. Signal Transduction Pathways

Signaling pathways play multiple roles in fungal development and pathogenicity. The G-protein regulated cyclic AMP signaling pathway, MAP Kinase cascades, and Ca^2+^/Calmodulin signaling pathways are highly conserved in phytopathogenic fungi and have been studied in *V. dahliae* in recent years. In the G-protein coupled cyclic AMP signaling pathway, extracellular signals are transmitted via membrane binding G-protein coupled receptors (GPCRs). G protein-mediated signaling is involved in the virulence, development, and hormone production of *V. dahliae* [68,69]. Gene knock out of the G protein β subunit gene (*VGB*) resulted in reduced virulence, increased microsclerotia formation and conidiation, and decreased ethylene production [68]. Mutants lacking *VdPKAC1*, the catalytic subunit of the cAMP-dependent protein kinase, are unable to form microsclerotia, produce high amounts of ethylene, and exhibit reduced virulence towards tomatoes [69]. Both VGB and VdPKAC1 regulate other signal pathway genes, including the MAP kinase, VMK11, and hydrophobin, VDH1 [68].

The mitogen-activated protein (MAP) kinase signaling pathway plays a major role in transducing external signals into the cell to invoke biological responses. In budding yeast, *Saccharomyces cerevisiae*, distinct MAP kinase pathways are required for mating, morphological changes, osmoregulation, and cell wall integrity [70,71]. In fungal pathogens, MAP kinase signal pathways are also involved in fungal pathogenicity [72,73]. Functional studies of the components of different MAP kinase pathways have confirmed the role of these proteins in the pathogenesis in *V*. *dahliae,* including a surface sensor (VdMsb) [74], an osmosensor (VdSho1) in the high osmolarity glycerol (HOG)-MAP kinase signaling pathway [75,76], a Hog1 MAP kinase (VdHog1) [77], VdPbs2, an upstream component of VdHog1 [78], Verticillium MAP Kinase 1 (Vmk1) [79], and MAPKKKs (VdSsk1, VdSsk2, and VdSte11) [80,81]. The deletion of these genes has shown that these MAP kinase cascades are involved in stress adaptation, plant root penetration, and microsclerotia formation in *V. dahliae*.

The Ca^2+^-calcineurin signaling pathway is conserved in eukaryotes and is involved in several biological processes including Ca^2+^ homeostasis and stress responses. In pathogenic fungi, the Ca^2+^-calcineurin signaling cascades are involved in host and environment adaptation, infectious structure formation, virulence, and antifungal drug resistance [82]. In response to external or internal signals, intracellular calcium concentrations increase, and calcium ions bind to the calcium-binding protein calmodulin, which in turn binds to and activates calcineurin, a serine-threonine phosphatase. Activated calcineurin dephosphorylates various target proteins, including the transcription factor Crz1 [82]. The Crz1 homolog in *V. dahliae*, VdCrz1, is required for cell wall integrity, microsclerotia development, and full virulence [83]. It has also been shown that reactive oxygen species (ROS) production elevates intracellular Ca^2+^ levels in specialized hyphal branch cells (hyphopodia) and activates VdCrz1, which induces penetration peg formation during early colonization in cotton roots [84].

The “target of rapamycin” (TOR) signaling pathway is also evolutionarily conserved in eukaryotes, and regulates cell growth, proliferation, and metabolism from yeasts to humans [85]. The putative components of TOR signaling pathways in *V. dahliae* (VdTOR) were recently identified [86]. When mycelia were treated with the TOR inhibitor rapamycin, growth and pathogenicity were significantly reduced and genes involved in various cellular processes, including ribosome biogenesis and cell wall degrading enzymes (CWDEs), were differentially downregulated. This suggests that VdTOR plays an essential role in hyphal growth, development, and pathogenicity [86].

During growth, development, and host infection in *V. dahliae*, these signaling pathways are under the control of complex regulatory networks that are governed by central transcriptional regulators. To date few are known in *V. dahliae*; however, Vst1 is involved in sporulation, melanin biosynthesis, and microsclerotia formation [87].

#### 2.3.2. Secondary Metabolism and Melanin Biosynthesis

Fungi produce an extensive array of secondary metabolites (SM) derived from several biochemical pathways, including polyketides, non-ribosomal peptides, terpenes, and indole alkaloids. These metabolites are mediated by the core enzymes known as polyketide synthases (PKSs), non-ribosomal peptide synthetases (NRPSs), terpene cyclases, and prenylation synthetases, respectively [88]. Polyketide synthase-nonribosomal peptide synthetase (PKS-NRPS) hybrid enzymes have also been identified and can be linked to the structurally diverse and complex SMs and further to their diverse biological activities in fungi [89]. Typically, genes involved in SM biosynthesis are clustered together [88]. In *V. dahliae,* 25 potential secondary metabolite gene clusters have been identified and 36% of those clusters were located in sub-telomeric regions close to the chromosomal end [90]. The phylogenetic and comparative genomic analysis suggested clusters in *V. dahliae* are involved in the biosynthesis of two putative siderophores, ferricrocin and triacetylfusarinine C (TAFC), 1,8-dihydroxynaphthalene (DHN)-melanin and fujikurin [90]. Melanin, a polyketide, is one of the most thoroughly studied SMs because it is directly linked to fungal cell wall stability and pathogenicity [75,81,91,92,93]. In *V. dahliae*, black melanin granules are heavily deposited in the cell wall of the survival structure, the microsclerotia [94]. Many of the genes in a melanin biosynthesis gene cluster are highly induced during microsclerotia formation [95]. Functional analyses of these genes have shown that both the central polyketide synthase VdPKS1 (VDAG_00190) gene and a transcription factor VdCmr1 (VDAG_00194) are required for melanin biosynthesis, with VdCmr1 being associated with the regulation of gene expression of VdPKS1 [91,92]. VdPKS1 is involved in *V. dahliae* virulence, conidiation, and ethylene production, even though microsclerotia production itself is not affected in the VdPKS1 mutant strain [92]. VdPKS1 is also regulated by MADS-Box transcription factor VdMcm1 (VDAG_01770), which is a key regulator in *V. dahliae* and is involved in melanin biosynthesis, conidiation, microsclerotia formation, and virulence [96]. VdMcm1 also controls PKS/NRPs hybrid-cluster gene expression. Deletion of Nag-1 in this cluster results in defects in growth, virulence, and melanin biosynthesis [97]. The Vayg1 gene, a homolog of Aayg1 from *Aspergillus fumigatus* (Aayg1), is also required for both melanin and microsclerotia production in *V. dahliae* [93].

#### 2.3.3. Cell Wall Degrading Enzymes and Carbohydrate Modifying Enzymes

Comparative genomics studies have revealed that *V. dahliae* has developed enhanced carbohydrate degrading machinery of potential value for weakening plant cell walls [44]. Polysaccharide lyase (PL) families, including PL1, PL3, PL4, and PL11, directly target different forms of pectin. Glycoside hydrolase (GH) families hydrolyze the glycosidic bond between carbohydrate compounds generated by PL and are significantly enriched in *V. dahliae* compared to other ascomycete fungi. *V. dahliae* has 30 proteins that contain the conserved carbohydrate-binding module 1 (CBM1), generally known as a fungal specific cellulose-binding domain. CBM1 is widespread in fungal enzymes, including PL proteins. *V. dahliae* has three CBM1-containing PL proteins [44]. Pectin degrading enzymes, which are highly secreted during fungal infection, play a key role in pathogenesis. Gene knock-out mutants lacking the pectin lyase genes *VdPL3.1* and *VdPL3.3* were unable to develop wilting symptoms in cotton [98]. High levels of pectin lyase activity occur during the compatible interaction between tomatoes and *Verticillium* spp. before disease symptoms appeared [99]. Furthermore, VdPEL1 triggers plant immunity responses and is involved in *V. dahliae* virulence. This implies that during infection, the pectin hydrolysis products may function as damage-associated molecular patterns (DAMPs) to elicit plant defense responses [100]. Similarly, *V. dahliae* cutinase, VdCUT11, acts as a virulence factor and can induce plant defense responses mediated by the leucine-rich repeat (LRR)-RLP/SOBIR1/BAK1 receptor complex in tobacco [101]. This response can be further suppressed by VdCBM1, a member of the carbohydrate-binding module family 1 (CBM1) in *V. dahliae* [101,102].

#### 2.3.4. Effector Proteins in *Verticillium dahliae*

Fungal effector proteins are typically secreted proteins that are associated with host determination and colonization of host plants [103]. In *V. dahliae*, about 700 proteins contain a signal peptide that guides the protein into the extracellular plant spaces. Typically, known effector proteins are small, with a high cysteine content in addition to a signal peptide. Studies have suggested *V. dahliae* contains ~150 small-secreted effector proteins that are less than 400 aa, with more than 4% cysteine content [44]. Recently, combining SignalP and EffectorP effector searching tools, we predicted about 200 core effector proteins among 18 sequenced *V. dahliae* genomes (Ingram et al., unpublished data). Additionally, *V. dahliae* isolates possess lineage-specific (LS) regions that contain predicted effectors; in many cases, such regions contain avirulence or virulence factors [74,78,103,104,105], including two currently known avirulence factors, Ave1 and Av2. Phylogenetic analyses have revealed that some of the LS genes were acquired by *V. dahliae* from other microorganisms, including *Fusarium* spp., through horizontal gene transfer [51,90,106].

The *Ve1* gene-mediated resistance against *V. dahliae* had been employed for many years in tomato breeding programs but elucidating the corresponding avirulence factor only became possible after the advent of modern molecular technologies, whole-genome sequencing, and transcriptome analysis. Comparative genome sequence analyses between avirulent (race 1) and virulent (non-race 1) *V. dahliae* isolates against tomato lines containing the *Ve 1* locus led to a 50-kb race 1 lineage-specific region being identified. Further gene expression profiling and transgenic expression led to the discovery of the identity of the small-secreted effector VdAve1 [52]. Recently, a similar approach was applied to identify another avirulence factor, VdAv2, which governs resistance in tomato lines which contain the V2 resistance locus [39]. Both VdAve1 and VdAV2 fall into the typical effector category; small cysteine-rich effector proteins located in lineage-specific chromosomal regions that are highly expressed during host colonization [52]. These characteristics could be particularly useful for the future discovery of new avirulence factors. In both cases, loss of recognition of these effectors by the host occurred through the deletion of DNA segments, rather than single nucleotide polymorphisms (SNPs) [39,52].

Chitin is a major structural component of the fungal cell wall. When a host plant is attacked by a fungal pathogen, chitin-degrading enzymes are released by the host into apoplastic space to release chitin oligomers, which activate pattern triggered immunity (PTI). These fungal chitin oligomers are recognized by lysin motif (LysM)-containing receptors in the plant membrane [107]. LysM effectors that also contain chitin-binding motifs are ubiquitous in phytopathogenic fungi and fungi that are pathogenic to mammals. These effectors function by sequestering fungal chitin fragments and preventing their recognition by host LysM receptors, blocking chitin-triggered plant immunity. In *V. dahliae*, the family of LysM effectors has expanded to contain six to seven LysM effectors [44]. Functional analysis of three core LysM effectors showed that they are not expressed during host colonization, nor are they involved in pathogenicity or fungal development. In contrast, a lineage-specific LysM effector (Vd2LysM) in the strain VdLs17 functions as a virulence factor [108,109]. Like LysM effectors, a secreted polysaccharide deacetylase (PDA1) in *V. dahliae* targets fungal chitin oligomers for successful fungal colonization. Rather than physically sequestering chitin, VdPDA1 converts chitin oligomers into chitosan and prevents activation of chitin-triggered immunity. VdPDA1 does not inhibit host chitinases activity, nor is it involved in fungal development [110].

#### 2.3.5. Genome-Wide Analysis of Host–Pathogen Interactions with *Verticiliium dahliae*

The development of large-scale transcriptomic, proteomic, and metabolomic technologies and availability of functional databases such as Gene Ontology (GO), Kyoto Encyclopedia of Genes and Genomes (KEGG) pathways, and the Plant Resistance Genes database (PRGdb), are providing opportunities to gain more detailed insight into pathogenicity and host defense responses.

In *V. dahliae*, genome-wide RNA-sequencing (RNA-seq) expression analyses have revealed important biological pathways during microsclerotia formation, including ubiquitin-mediated protein degradation and melanin biosynthesis [95,111]. Gene expression is regulated not only at the mRNA level but also through alternative splicing because about 50% of intron-containing genes are possibly regulated by alternative splicing [112]. Other gene expression studies have shown that *V. dahliae* responds more strongly to root exudates from a susceptible cultivar than tolerant and resistant cultivars, as evidenced by increased gene expression for hydrolase activity; particularly genes involved in hydrolyzing O-glycosyl compounds at the early stages of the interaction [113]. Differential root exudate profiles were also associated with tomato rootstock grafted to eggplant scions, compared to non-grafted eggplants, and this was associated with suppression of mycelial growth and enhancement of mycelial growth, respectively [114]. Suppression of mycelial growth is associated with delayed onset of *V. dahliae* colonization and symptom development.

Defense-related gene expression responses are activated by various abiotic and biotic stresses in plants. In tomatoes, physical wounding induces defense-related genes, including the *Ve1* gene, in both susceptible and resistance lines [115]. During the compatible interaction between tomatoes and *V. dahliae*, compared to non-inoculated control plants, 1953 significantly differentially expressed genes (DEGs) were identified in the root samples two days after inoculation. Most of the DEGs were linked to phenylpropanoid metabolism and plant-pathogen interaction pathways [104]. Comparative proteomic and metabolomics of compatible and incompatible interactions with *V. dahliae* provided differential profiles in tomato stem tissues. During the incompatible interaction between Beefsteak (Ve+) tomato and Le1087 (race 1) *V. dahliae*, higher levels of phenolic compounds responsible for plant defense mechanisms and enzymes involved in plant defense responses, including phenylalanine ammonia-lyase (PAL) and lignin biosynthesis, were significantly induced [116]. These resistance-related responses were consistent across the entire host plant because similar groups of genes were induced in the *V. dahliae*-inoculated root tissues during incompatible interactions in a separate study [117]. Similarly, transcriptional profiles of sunflowers infected with *V. dahliae* revealed that a large group of genes responsible for plant defense is induced in both resistant and susceptible hosts, with higher induction in resistant host lines compared to susceptible ones. Genes involved in hypersensitive responses and the salicylic and jasmonic acid-mediated signaling pathways are linked to *V. dahliae* resistance [118].

RNA-seq analyses upon *V. dahliae* infection have confirmed previously known common resistance-associated biological pathways in host plants. Temporal transcriptional analysis from *V. dahliae* inoculated *Arabidopsis thaliana* revealed 13,916 differentially expressed genes (DEGs), including 401 transcription factors, compared to mock-treated plants [104]. Gene ontology (GO) functional classification of DEGs identified a total of 2308 genes involved in the stress response, which were subcategorized into 453 DEGs associated with defense responses, 369 with the regulation of the plant-type hypersensitive response, and 358 with the defense response to fungi. Pathway analysis of DEGs showed that the genes involved in the biosynthesis of secondary metabolism are greatly enriched, and a group of genes related to plant-pathogen interaction, plant hormone signal transduction, phenylalanine metabolism, and flavonoid biosynthesis was highly enriched. Genes (413) involved in the SA hormone signaling pathway and 404 genes involved in JA signaling were differentially expressed during the infection process [104]. Similar gene expression patterns were exhibited during the interaction between the wild-type resistant eggplant and *V. dahliae*: 17,645 DEGs were identified, and genes involved in the phenylpropanoid pathway, lignin biosynthesis, and plant hormone signal transduction and genes encoding pathogenesis-related proteins (PRs) and transcription factors were induced during this incompatible interaction [119].

Currently, cross talk analysis between *V. dahliae* and tomato gene expression remains challenging because the majority of the transcripts from infected tissue samples are mapped to the host genome; less than 1% of the reads mapped to fungal genomes [120]. Likewise, only a few proteins from infected plant tissue have been mapped to the *V. dahliae* proteome [116]. This lack of fungal information greatly impedes attempts to link fungal gene/protein patterns with corresponding host responses.

## 3. Current Measures and Limitations of Verticillium Wilt Management

### 3.1. The Genetic Basis of Plant Disease Resistance

Broadly, the interactions between tomatoes and *V. dahliae* result in three states: (i) susceptibility (compatible interaction), (ii) resistance (incompatible interaction), and (iii) tolerance (intermediate interaction). In susceptibility, the fungus proliferates systemically throughout the plant, leading to symptom expression and disease development. Disease resistance is further grouped into qualitative and quantitative resistance.

#### 3.1.1. Qualitative Disease Resistance

As described above, resistance to *V. dahliae* race 1 is conferred by a single dominant Ve locus and incorporated into tomato breeding programs [24]. The Ve locus contains two closely linked inversely oriented genes, Ve1 and Ve2, which are mapped onto tomato chromosome 9 [31]. Intriguingly, only Ve1, but not Ve2, confers resistance to *V. dahliae* in tomatoes [121]. The interfamily transfer of Ve1 to *Arabidopsis thaliana* provided fully functional Verticillium wilt resistance [122]. Comparative genomic analysis of race 1 identified an effector, Ave1, which is a small-secreted protein with four cysteines that contributed to virulence in tomato plants lacking Ve1 [30]. The Ve locus encodes the extracellular leucine-rich repeat receptor-like protein, a class of R protein, and triggers effector-triggered immunity in the host [31].

#### 3.1.2. Quantitative Disease Resistance (QDR)

Quantitative disease resistance (QDR) is conditioned by multiple genes or the quantitative trait loci (QTL) of small effects and may interact with the environment [123]. Generally, QDR is race non-specific and provides partial resistance, which reduces pathogen multiplication, plant colonization, and disease severity [124]. The effects of QDR are often additive and more durable than R gene-mediated resistance [124]. We have found variation in the level of resistance to *V. dahliae* races 2 and 3 in the tomato germplasm; however, the biological and molecular basis of resistance to these races in tomatoes is still unclear, and needs further research (Ingram et. al, unpublished data).

#### 3.1.3. Plant Tolerance

Hosts that are tolerant have reduced symptoms and produced higher yields compared to susceptible ones [125,126]. Host tolerance can be quantified using pathogen biomass, disease severity, and yield impacts in the host cultivar [126]. In *V. dahliae*, hyphae colonize internal tissues and spread systemically [125]. Vascular wilt severity is often assessed using a disease index [127,128] or percentage of chlorosis and necrosis of leaves [125], while plant growth is quantified by measuring stem height [125,128] or by fresh weight [127]. Although host tolerance to *V. dahliae* was controlled by polygenes in cotton and potatoes [129,130], a single dominant gene, *VET,* was found to enhance tolerance in *Arabidopsis thaliana* L. [128]. The use of tolerant cultivars as rootstocks or sources of resistance in breeding programs may be a productive pursuit as a component of an integrated pest management (IPM) strategy to manage Verticillium wilt.

### 3.2. Grafting as a Measure to Combat Verticillium Wilt

Grafting tomatoes has been documented as a tool to manage important soilborne pathogens [131,132]. However, long-term success has not been found using a diversity of rootstocks to manage Verticillium wilt of tomato. As expected, *V. dahliae* race 1 resistance provides a high degree of protection to susceptible scions in the regions where race 1 predominates [131,132,133,134,135]. Likewise, it is reasonable to anticipate that race 2 resistance would protect susceptible scions in the field where race 2 predominates; currently, there is only laboratory and greenhouse evidence that race 2 resistance protects tomato plants [37,38]. Since pathogen races can only be discerned as resistance genes are deployed, the durability of any single gene is uncertain. For example, the discovery of race 2 resistance enabled elucidation that race 3 (non-race 1 and non-race 2 isolates) is widespread throughout Japan and North America, ([35], Ingram et al., unpublished data]). The long-term success of any given rootstock with race-specific resistance will rely on widespread pathogen screening in regions where those rootstocks are deployed.

Varieties developed from interspecific hybrids between *S. lycopersicum* and *S. hirsutum* may protect against the wilting symptoms of *V. dahliae* [135]. The interspecific tomato hybrid rootstock “Beaufort” reduced disease in eggplant scions in field trials [131], although the reduction in disease and subsequent yield increases may simply have been the result of higher vigor [135,136], or possibly a form of tolerance as discussed above. Future studies will need to address whether increases in plant health from rootstocks are due to resistance or vigor.

### 3.3. Chemicals in Use for Verticillium Wilt Management

With the phase-out of methyl bromide due to environmental concerns, a search for effective alternate soil fumigants and other chemicals, and biologically based methods to manage *V. dahliae,* continues. Even though some alternative biological methods have been identified (see below), no new fungicide is currently available for use against *V. dahliae.* The most common fumigants, 1,3-dichloropropene (1,3-D), trichloronitromethane (chloropicrin), 3,5-dimethyl-(2H)-tetrahydro-1,3,5-thiadiazine-2-thione (dazomet), dimethyl disulfide (DMDS), sodium and potassium- N-methyldithiocarbanate (metam sodium, metam potassium) and their combinations, have been widely evaluated for their efficacy to manage soilborne pathogens, including *V. dahliae* [137,138,139]. These fumigants, singly (not 1,3-D) or in combination, are effective in reducing wilt incidence and *V. dahliae* microsclerotia in soil [138,140,141]. In an experiment using bell peppers, the use of chloropicrin at 30 and 40 g m^-2^, applied by drip irrigation, reduced Verticillium wilt disease incidence significantly [142]. The reduction in the disease progress rate was better than when using dazomet at 40 g m^-2^ in this study. The systemic fungicide, thiophanate-methyl, was effective against *V. dahliae* in potatoes, but only partially and when disease pressure was not high [143]. In another experiment with chrysanthemums, DMDS, chloropicrin, and metam sodium had similar effects in terms of significantly reducing Verticillium wilt incidence compared to the control [138]. When fumigants (DMDS, chloropicrin, 1,3-D) were used alone, a higher dosage was required for *V. dahliae* and *Meloidogyne incognita* suppression, but when any two of these fumigants were combined, the lower dosage was effective [144]. Dazomet was also reported to promote phosphorus mineralization and allowed crops to absorb and use phosphorus [145].

Fumigants have sometimes been integrated with non-chemical approaches, such as using resistant rootstocks and bio fumigants, to improve disease control, reduce the use of chemicals, and protect the soil environment. When chloropicrin was alternated with bio-fumigation (fresh chicken manure + wheat straw), the nutrient availability in soil was improved and increased the strawberry marketable yield and microbe genetic diversity in the soil [146].

However, soil treated with some of these fumigants has sometimes shown a detrimental effect on the soil biochemical properties and microbiome. For instance: chloropicrin inhibited conversion of ammonia to nitrite in five different soil types [147], and chloropicrin and dazomet treatments lowered microbial activities and soil microbiome biomass, decreased alkaline phosphatase harboring microbes, and also resulted in different microbiomes as compared to those of anaerobic soil disinfestation (ASD) treatments [137,148]. However, a study with metam-sodium showed a mixed effect, where it inhibited substrate-induced respiration, microbial biomass nitrogen, and accumulated ammonium ions in the soil in short term, but reduced the population of bacteria and fungi in the soil and shifted soil bacterial population to plant growth-promoting bacteria and biodegrading bacteria [149]. The negative impact of these chemical fumigants on the soil physicochemical properties and microbiome has provided an impetus to advance the science of non-chemical alternatives to manage *V. dahliae*.

### 3.4. Biocontrol Agents and Biologicals to Manage Verticillium Wilt

Biocontrol agents (BCAs) are microorganisms that are used to manage several pests, including insects and plant pathogens of agriculturally important crops, either by reducing pathogen inoculum or its ability to cause disease [150], while biologicals are products obtained from living organisms. Biocontrol is a tool in an integrated management strategy that is environmentally friendly and is viewed as a potential alternative to chemical pesticides, to prevent their side effects [150]. Parasitism, competition for nutrients and space, antibiosis, and induced systemic resistance (ISR) are major biocontrol mechanisms [150,151]. The desirable traits of BCAs and their uses against Verticillium wilt have been discussed elsewhere [152]. However, several recent studies have broadened the scope of BCAs with some new candidates within the well-established genus of *Bacillus, Pseudomonas,* and *Trichoderma,* and beyond (Table S2). Currently, studies involving BCAs not only look for organisms with antagonistic properties, but using the available genetic tools, their mode of action, genes, proteins, and the metabolites involved have also been characterized. For instance, in *Bacillus velezensis* AL7, a biocontrol agent isolated from cotton soil that synthesizes antifungal antibiotics, 3706 protein-coding genes, 86 tRNAs, and 27 rRNAs were predicted which can help identify the candidate genes involved [153]. Similarly, transcriptomic analysis of *Trichoderma atroviride* T11 identified the *cpa1* gene, whose increased level of expression and protease activity has been associated with higher antifungal activity against *V. dahliae* V-138I [153,154]. These findings open avenues for further understanding of these BCAs to increase their efficacy for commercialization.

In some cases, mixing different BCAs or their extracts among themselves, or with organic amendments, has provided better management of *V. dahliae* [150,155,156], since mixing increases the biological activities of microbes and/or their extracts. Little information is available regarding the use of biologicals to manage *V. dahliae,* but oils, derivatives, and extracts from medicinally important plants and some algae are being tested, with encouraging results [157,158]. However, more research is required to explore new sources and mechanisms of action before further use. Although potential applications of BCAs against *V. dahliae* have emerged, most of the research on BCAs has been conducted in vitro or greenhouses under controlled conditions. A major problem with the widespread use of BCAs is their inconsistent efficacy when tested under field conditions. However, some BCAs have shown promising results when experimented within the fields against *V. dahliae* with olives [159] and cotton [160], and combining BCAs with different modes of action has offered some efficacy [150,161]. Ensuring the long-term viability of BCAs and biologicals for storage is another problem that needs to be taken into consideration for commercialization, and their practical application in the field.

### 3.5. Organic Amendments

For soilborne pathogens such as *V. dahliae*, chemical-based suppression has not proven sustainable and the use of organic amendments (OAs) has been explored to design suppressive conditions to limit pathogen infestation levels or onset of disease. OAs include materials that are worked into the soil or applied on the surface to improve the physical properties of the soil, and by fostering living microorganisms that are present in the soil, to directly or indirectly impact disease incidence [150]. Some examples of OAs used to manage *V. dahliae* in various crops include plant and animal-based composts and manures; green manure/cover crops; and other industrial co/by-product wastes (Table S3).

Composts not only add organic matter to the soil but also serve as a reservoir to foster a microbiome that can protect crops through increased soil microbial activities against soil-borne pathogens [162]. Compared to animal-based amendments (dairy and horse manure), plant-based amendments can impact pathogen success due to deleterious chemicals introduced from the plants, in addition to supporting beneficial microbial activities [163]. Cover/green manure crops are rotated with main crops to cover the soil surface and can improve the physical, chemical, and biological properties of the soil [164]. Furthermore, they can be incorporated into the soil to suppress soil-borne pathogens [165]. Crops in the Brassicaceae family are a good example of green manure often used in crop rotation to reduce soil-borne pests and pathogens. They are rich in glucosinolates, the precursors of isothiocyanates that produce volatile sulfur compounds, known for their fungicidal, nematocidal, and allelopathic properties through bio-fumigation [165]. When green manures are polyethylene-covered, the toxic effects on pathogens are greater compared to their application to open soil.

As with biocontrol agents, a single OA may not provide sufficient pathogen suppression; when applied as a mix of OAs or with biocontrol agents, efficacy was better [150,166,167]. However, factors such as the type of amendment, the lack of standardization of application rates, the inconsistency in their efficacy, and phytotoxic effects of released toxic compounds on crops limit the applications of OAs for disease management [168] and deserves further investigation.

### 3.6. Anaerobic Soil Disinfestation (ASD)

Anaerobic soil disinfestation (also known as reductive soil disinfestation or biological soil disinfestation) is an organic amendment-based pre-plant soil-borne disease management tool [169,170]. For ASD, the soil is first amended with a carbon source, irrigated to field capacity to fill soil pore spaces with water, and covered with an impermeable plastic tarp or surface-sealed using other methods, to limit gas exchange for several weeks to complete the ASD treatment [171,172]. Some examples of carbon sources from recent studies of ASD used in various crops include rice-bran, molasses, ethanol, and others (Table S4).

ASD has proven effective against a wide range of soil-borne pathogens in many different cropping systems, however, the efficacy against a target pathogen depends on the carbon-source used, tarp type, soil type, soil microbiome, and soil temperature retained during ASD [170,172]. Ebihaha and Uematsu [173] tested the survival of three strawberry pathogens under anaerobic conditions and found that *V. dahliae* did not grow under anaerobic conditions at 22.5 °C, indicating that anaerobic conditions obtained during ASD can have a fungistatic effect on *V. dahliae*. ASD treatments also induced changes in soil microbial communities and increased the soil microbial activity, and the populations of Bacteroidales, Clostridiales, Selenomonadales, Enterobacteriales, Sphingobacteriales, Bacillales, and Burkholderiales that antagonize plant pathogens [137,169,174]. The change in the bacterial communities and composition increased denitrification and nitrogen fixation and produced organic acids that influenced disease suppressiveness [175]. Optimizing the carbon source for ASD can improve the effectiveness of ASD and affordability for growers [169]. An economic analysis of ASD for open-field fresh-market tomato production using molasses and composted poultry litter showed that ASD requires higher labor costs for land preparation and treatment application, but the yield increase from ASD treatment was enough to cover the increased labor cost [176]. Similarly, in the studies with strawberries, where different carbon sources of ASD were compared to chemical treatments (PicChlor 60), the net return and marketable yield were either similar or increased due to ASD (e.g., rice bran) [140,177]. Even though the issues related to efficacy, cost, and standardized application rates of the carbon source need attention [169,170,172], the results obtained from ASD studies are encouraging, and it is gaining in popularity.

Most of the current studies on utilizing non-chemical-based approaches to manage *V. dahliae* have focused on cotton, olives, strawberries, and eggplants, and little information is available for tomatoes. Hence, experimenting with the potential BCAs, OAs, and carbon sources trialed for other crops with the tomato-*V. dahliae* pathosystem could help identify the candidates that may most benefit tomato growers.

## 4. Novel Approaches and Future Directions

### 4.1. Exploiting Plant Microbiomes: Application to the Verticillium dahliae System

Microbiomes are composed of numerous individuals (e.g., bacteria, fungi, actinomycetes, viruses, and protists) of diverse species [178]. All tissues of a plant harbor microbiomes, including the roots, leaves, shoots, flowers, and seeds. Based on the association with habitats in the host plants, microbiomes are classified as the rhizosphere, phyllosphere, and endosphere microbiomes [179]. The rhizosphere is a rich and soil-derived microbial diversity zone, which is influenced by plant roots through the rhizodeposition of exudates and mucilages [180]. Although a few reports have been described around their potential usefulness [179,180,181,182], little is known about how these microbiomes play beneficial roles in tomato–*V. dahliae* interactions. Recently, phyllosphere microbes residing on the leaf surface were found to be mainly epiphytes, and are influenced by leaf structures such as veins, hairs, and stomata [183]. Tomato rootstocks have differential impacts on tomato scion phylospheres [184], however; there are no published reports on tomato × *V. dahliae* interactions. Endosphere microbiomes reside within the intracellular apoplast and in the xylem vessels, which may enter through natural breaks in the roots and root tips and translocate to the aerial parts of the plant [179]. Endosphere microbes are typically latent and non-pathogenic and can influence host metabolism and plant immunity [185]. The antagonistic activity of *B. amyloliquefaciens* from different cultivars and regions against the olive-pathogenic *V. dahliae* also showed a close functional relationship [186]. Interestingly, an endophytic, non-pathogenic *Fusarium solani* (strain CEF559) also conferred protection against *V. dahliae* [160]. To date, the microbiomes of tomato plants growing under field conditions remain poorly characterized, and many of the roles and interactions of diverse disease-resistant rootstocks and field environments remain to be elucidated. We hypothesize that many of the benefits of rootstocks are mediated by soil and rhizosphere microbiomes and that intra- and inter-specific genetic variation can impact the structure and composition of the microbial community and suppress *V*. *dahliae* to enhance plant health. Moreover, plant root exudates may contain signal molecules that may influence species composition in the rhizosphere. Increasing evidence supports the hypothesis that the association between grafted tomato rootstocks and rhizosphere microbiomes can improve plant growth [187]. Beneficial microbiomes also activate immune systems, such as induced systemic resistance (ISR) [188] and systemic acquired resistance (SAR), to plant pathogens [189]. Mapping of genetic populations and innovative grafting experiments can be conducted to test ecological hypotheses and devise prescriptive approaches to manage microbiomes to suppress Verticillium wilt problems in tomatoes.

**Figure 3:**
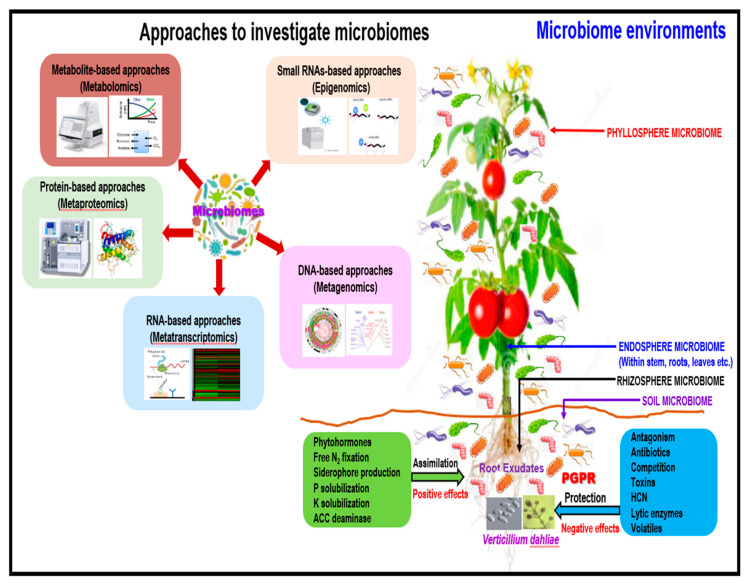
A proposed schematic illustration of the plant microbiome impact on *Verticillium dahliae* protection, and crop productivity, and crop yield. Generation concepts and mechanisms of defense, and novel approaches, were adapted and modified from previous publications [178,181,182,183,184,185,186,187,188,190,191].

Mapping populations and grafting resistant scions onto resistant rootstocks can be used to test the ecological hypotheses and for the discovery of new molecules or compounds in the rhizosphere and phyllosphere microbiomes using new approaches (Figure 3).

### 4.2. Novel Molecular and Genomic Approaches to Enhance Verticillium Wilt Resistance

Although conventional breeding plays an important role in developing and testing several tomato lines in the field, traditional improvement methods are time-consuming and troublesome. However, breeding efforts to exploit the genetic variability in the cultivated and wild relatives of tomatoes and utilize resistance to *V. dahliae* and other pathogens in tomato breeding programs, have been achieved with some success. For example, wild tomato relatives such as *L. pimpinellifolium*, *L. peruvianum*, and *L. hirsutum* have been utilized as sources of resistance to develop segregating breeding populations to test against *V. dahliae* and other pathogens [192]. Other populations that have been developed include recombinant inbred lines (RILs), near-isogenic lines (NILs), and multiparent advanced generation inter-cross (MAGIC) populations [192,193]. Although conventional breeding has been successfully used to improve yield and quality and meet consumer requirements, the introgression of *R* genes or QTL and examination of large populations is time-consuming and labor-intensive [194]. Molecular markers such as cleavage-based cleaved amplified polymorphic sequences (CAPS), kompetitive allele-specific PCR (KASP), simple sequence repeats (SSR), single nucleotide polymorphisms (SNPs), and InDels [195] have been developed and used to locate and tag genes or QTLs for disease resistance and other traits in tomatoes via marker-assisted selection (MAS) [196,197,198]. Whole-genome resequencing approaches, such as QTL-seq [199], genetic mapping and mutant identification (MutMap) [200], bulked-segregant analysis based on RNA-seq (BSR-seq) [201], specific locus amplified fragment sequencing (SLAF-seq) [202], and genome-wide association studies (GWAS) [203] have also been utilized to identify candidate genes or markers linked to the genes of interest in tomatoes.

Plants have developed advanced defense mechanisms to protect themselves against pathogens [204]. Plasma membrane-bound and intracellular immune receptors initiate innate defense responses upon the perception of pathogens either directly interacting with pathogen-derived immunogens or indirectly interacting by monitoring modifications of host targets incurred by pathogens [204,205]. Plant-derived antimicrobial peptides and other compounds such as *FLS2*, *LecRK-VI.2*, *EFR*, *CERK1*, *Ve1,* and *PERPs* all belong to the receptor-like kinases (*RLKs*) [206] that inhibit pathogen virulence [207,208]. In contrast, plant pathogens have also evolved some offensive tools to overcome host immune responses. These weapons include cell-wall degrading enzymes (CWDEs) that disintegrate the plant cell wall for successful colonization and establishment [209], and secretion systems to deliver effectors into the host cytoplasm to suppress host defense and promote colonization [210,211]. The advances in biotechnological innovations and development of next-generation sequencing technologies, and some aspects of the host-microbe, provide opportunities to greatly enhance functional investigations and promote the deployment of useful disease resistance genes [212], which portends a promising future in managing Verticillium wilt in tomatoes.

### 4.3. Identifying Quantitative Disease Resistance (QDR) and Pyramiding for Broad-Spectrum and Durable Resistance

**Figure 4:**
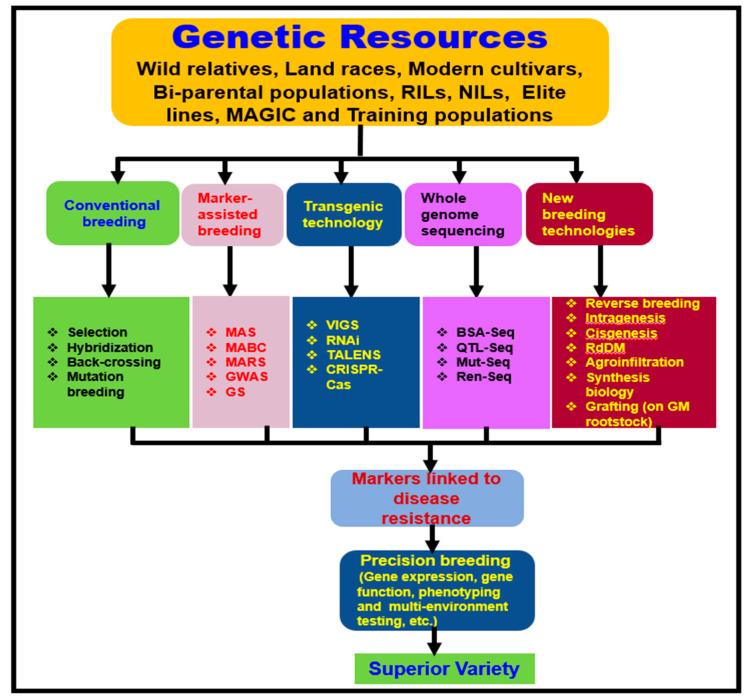
A proposed flow-chart to develop Verticillium wilt-resistant tomato varieties by genome-based approaches and new plant breeding techniques adapted and modified from previous publications [192,193,194,195,196,197,198,199,200,201,202,203,205,212,221,222,223,224,225].

Durable disease resistance refers to a resistance that remains effective over a prolonged period [213]. A better understanding of pathogen biology, population genetic structure, disease epidemiology, and mechanisms of genetic variability can support the prediction of the durability of disease resistance [214]. To develop broad-spectrum and durable resistance, a gene pyramiding (also known as gene stacking) strategy has been used to deploy multiple *R* genes into a single cultivar simultaneously [215]. For instance, resistance to multiple races of rice blast and bacterial blight was accomplished by pyramiding genes through MAS [216,217]. Similarly, broad-spectrum resistance to late blight pathogen was achieved by molecular stacking of three *R* genes on two separate occasions, (*Rpi-sto1*, *Rpi-vnt1.1*, and *Rpi-blb3*) [218] and (*RB*, *Rpi-blb2*, and *Rpi-vnt1.1*) [219], at a single genetic locus in potatoes using *Agrobacterium* transformation [220]. These new advancements offer an opportunity to rapidly identify several small effective alleles through genomics-enabled new breeding approaches [123,212] and stack them for broad-spectrum resistance to *V. dahliae* in tomatoes (Figure 4).

### 4.4. Exploring and Exploiting the Intracellular Immune Receptors

Resistance gene enrichment sequencing (Ren-Seq) [226,227] has been employed to identify regulatory elements and nucleotide-binding leucine-rich repeat (*NRL*) family proteins from uncharacterized germplasms [51]. Recently, Ren-Seq with single-molecule real-time (SMRT) has been utilized successfully to rapidly identify and clone anti-potato late blight *NLR* genes from wild potatoes [228], and four stem rust (*Sr*) genes for resistance to *Puccinia graminis* f. sp. *tritici* from wild accessions (*Aegilops tauschii* sp. *strangulata*) [229]. Two stem rust *NLR* genes, *Sr22* and *Sr45,* from hexaploid bread wheat, have been discovered, and these genes conferred resistance to multiple races of stem rust pathogen [230]. Ren-Seq is a potential novel method to rapidly uncover novel *NLR* genes for resistance to races 2 and 3 of *V. dahliae* from wild tomato species and utilize them in plant breeding programs.

### 4.5. Modulating MicroRNAs and Improving Plant Disease Resistance

Plants carry two major classes of small RNAs: microRNA (miRNA) and small interfering RNA (siRNA), which are endogenous, single-stranded non-coding RNA molecules (21–24 nucleotides in length) that combine with complementary sequences in target messenger RNAs (mRNAs) [231]. The RNA interference (RNAi) technique has been used to suppress the expression of a gene by the host- or the pathogen-induced gene [232]. Extensive studies have demonstrated that miRNAs play important roles in plants, particularly in tolerance to abiotic and biotic stresses [233,234]. Available evidence suggests that miRNAs also play important roles in plant immune responses [235,236,237]. For example, miR393 has been implicated in pathogen-associated molecular pattern-triggered immunity (PTI) [237]. miRNAs are considered master regulators of the *NLR* gene family [238,239,240]. Importantly, the miR482-mediated silencing cascade in *Arabidopsis,* cotton, potatoes, and eggplants enhanced plant defenses against *V. dahliae* [240,241,242]. Two *V. dahliae* genes, *Clp-1* (encodes a Ca2+-dependent cysteine protease) and *HiC-15* (encodes an isotrichodermin C-15 hydroxylase), were targeted by miR166 and miR159, respectively, and silencing of these two fungal virulence genes mediated resistance to *V. dahliae* [243]. As a result, the modulation of miRNAs by RNA silencing [244] offers a powerful strategy to improve our understanding of tomato-*V. dahliae* interactions, and to enhance plant defenses.

### 4.6. Harnessing Gene-Editing Technologies

More recently, genome-editing based on CRISPR-Cas9 (clustered regularly interspaced short palindromic repeats/CRISPR-associated protein 9) technologies have revealed a breakthrough for miRNA fine-tuning [245]. In this process, Cas9 protein (an RNA-guided nuclease) can be cleaved at a specific desired sequence on the substrate viral DNA or RNA, generating DNA double-strand breaks that usually result in gene silencing due to their degradation [246]. CRISPR-Cas9 and -Cas13a mediated single or multiple protein-coding gene knockouts have been developed and utilized to engineer resistance to DNA or RNA plant virus diseases [205]. The CRISPR-Cas9 genome-editing platform has also been employed to enhance resistance to *V. dahliae* in cotton. The indels of the *Gh14-3-3d* gene (signaling receptor proteins) were generated in the At and Dt sub-genomes of tetraploid cotton (*Gossypium hirsutrum),* and transgene-clean edited T2 plants showed enhanced host resistance to *V. dahliae* [247]. Using the CRISPR-Cas9 system, multiple genes—*lig1*, *ms26*, and *ms45*—were stacked in a single chromosomal location in corn [248]. Other major genome editing and new plant breeding techniques (NPBTs) that have been developed are homologous recombination (HR), meganucleases (MNs), zinc finger nucleases (ZFNs), transcription activator-like effector nucleases (TALENs), pentatricopeptide repeat proteins (PPRs), oligonucleotide-directed mutagenesis (ODM) cisgenesis, and intragenesis [212,221]. RNA-directed DNA methylation (RdDM), reverse breeding, genetically modified rootstock (GMO), agro-infiltration, and synthetic genomics have also been employed to improve crop varieties for sustainable production [212,221,222,223,224]. Although these methods have been utilized successfully in other crops, the utility of these novel genomic and reverse genetic tools to enhance resistance to *V. dahliae* in tomatoes needs to be explored.

## 5. Concluding Remarks

*V. dahliae* survives in the soil for an indefinite period. Importantly, this fungal pathogen has a wide host range (>400 host plants), which makes it difficult to improve host resistance. This review sought to provide recent information on *V. dahliae*, its importance in tomato production, molecular mechanisms involved in fungal pathogenicity, and an overview of current management tactics. The recent reports on the discovery of race 3 *V. dahliae,* and predictions of about 200 core effector proteins and others in lineage-specific regions, has opened up many opportunities for downstream research to elucidate the mechanisms and genes involved. For Verticillium wilt management, several non-chemical methods are being explored due to the reduction in the number of available chemical alternatives, and their harmful impacts on the environment and human health. More recently, studies involving biocontrol agents, organic amendments, and anaerobic soil disinfestation to manage *V. dahliae* have increased substantially; however, their efficacy for use in the field needs additional optimization. Recent advances in molecular and sequencing technologies have provided novel strategies for disease management, informing the future direction of implementation. For genetic resistance, the *Ve1* gene had been identified and deployed in tomatoes to manage race 1, but it was defeated due to the evolution of non-race 1 strains. A recent study suggests that resistance to *V. dahliae* race 2 in tomatoes is also conferred by a major gene. However, the presence of partial resistance and tolerance to *V. dahliae* is predicted in tomatoes, as variation in the resistance to race 2 and 3 has been observed in the tomato germplasm when tested under laboratory, greenhouse, and field conditions. The molecular mechanisms underlying disease resistance and the genes associated with this resistance are still unknown. Additionally, recent findings suggest the exploitation of microbiomes can enhance resistance and protect crops from pathogen invasions. Microbiomes associated with tomato–*V. dahliae* interactions have not yet been fully characterized, and future investigations are necessary. Importantly, novel molecular and genomic breeding approaches and genome editing tools are available for a better understanding of the mechanisms of resistance to *V. dahliae,* and the development of improved and enhanced disease-resistant tomato cultivars.

## Supplementary Materials

The following are available online at https://www.mdpi.com/2223-7747/9/11/1622/s1, Table S1: Disease compatibility of specific combinations of race-specific secreted effectors (Ave1 and V2) on resistant cultivars of tomatoes containing different combinations of the race 1 (Ve1) and race 2 (V2) resistance phenotypes, Table S2: Biocontrol agents (BCAs) and biologicals with biocontrol activity against *Verticillium dahliae*, Table S3: Some examples of organic amendments (OAs) with suppressiveness against *Verticillium dahliae*, Table S4: Examples of sources of carbon from recent studies for anaerobic soil disinfestation of soil-borne pathogens including *Verticillium dahliae*. References [137,140,153,154,155,156,157,158,159,160,161,163,164,166,167,169,174,175,224,249,250,251,252,253,254,255,256,257,258,259,260,261,262,263,264,265,266,267,268,269,270,271,272,273,274,275,276] are cited in the supplementary materials.
