# Supplementary material for: Opportunities and Challenges in Studies of Host-Pathogen Interactions and Management of Verticillium dahliae in Tomatoes
Source: Plants (Basel). 2020 Nov 22;9(11):1622. doi: 10.3390/plants9111622 (PMC7700276; doi:10.3390/plants9111622)
Supplement: Supplementary file 1 [file plants-09-01622-s001.pdf]

plants

MDPI

Table S1. Disease compatibility of specific combinations of race-specific secreted effectors (Ave1 and V2) on resistant cultivars of tomatoes containing different combinations of the race 1 (Ve1) and race 2 (V2) resistance phenotypes.

		Resistance combination			
**Race (isolates)**	**Race gene**	**Ve1-/V2-**	**Ve1+/V2+**	**Ve1+/V2-**	**Ve1-/V2+**
Race 1/2 (Le1087, JR2)	Ave1+/Av2+	Disease	Resistant	Resistant	Resistant
Race 2 (To22, Ca36)	Ave1-/Av2+	Disease	Resistant	Disease	Resistant
Race 3 (HoMCF, KJ14a)	Ave1-/Av2-	Disease	Disease	Disease	Disease
Race 1/3 (Vdp4)	Ave1+/Av2-	Disease	Resistant	Resistant	Disease

Table S2. Biocontrol agents (BCAs) and biologicals with biocontrol activity against *Verticillium dahliae*.

**Biocontrol organism**	**Source**	**Mechanism of action /efficacy**	**References**
Acinetobacter calcoaceticus FS339	Root (Tomato)	Reduced mycelial growth, reduced disease index, increased chitinase, siderophores, protease activity	[249]
Bacillus axarquiensis TUBP1 protein	Rhizosphere (Cotton)	Anomalies in fungal hyphae and conidia, reduced spore germination, targeted plasma membrane of *V. dahliae*	[250]
Bacillus spp.	Composted ginned cotton residue	Reduce disease severity and wilt symptoms, antibiosis	[161]
Bacillus thuringiensis	Rhizosphere (Tomato)	Mycolitic chitinases, polypeptide synthetases, bacillibactin	[251]
Bacillus velezensis AL7	Soil (Cotton)	Synthesize antifungal antibiotics	[153]

**Biocontrol organism**	**Source**	**Mechanism of action /efficacy**	**References**
Bacillus velezensis C2	Endophyte (Tomato)	Reduced disease incidence, antifungal activities (synthesis of lipopeptides, presence of volatile metabolites, produce lytic enzymes	[252]
Bacillus velezensis OEE1	Endophyte (Olive)	Reduced fungal growth (92%), disease severity index, percentage of dead plants, and microsclerotia density	[159]
Burkholderia gladioli pv. *agaricicola*	-	Reduced mycelial growth and disease incidence	[253]
Cell-free mix of *Sphingobacterium* A1 and *Bacillus tequilensis* C-9 (1:9)	Rhizosphere (A1 from *Resina ferulae* and C-9 from cotton)	Reduced spore production (97.8%), germination (100%), and virulence protein of *V. dahliae*	[155]
Chaetomium globosum CEF-082	Endophyte (Cotton)	Biocontrol effect 59%, Regulate multiple metabolic pathways, induce defense responses	[254]
Crystoseria myriophylloides, Laminaria digitata, Fucus spiralis	Algal extracts	Reduced disease severity, Increase defense response in tomato due to polyphenol oxidases, peroxidases	[255]
The essential oil from *Thymus* spp.	Thyme	Inhibition of mycelia and microsclerotia; disease reduction	[157]

Biocontrol organism	Source	Mechanism of action /efficacy	References
Fusarium solani CEF559	Endophyte (cotton)	Reduced colony growth (75%) and sporulation (80), PR genes and genes in lignin metabolism pathway upregulated, greenhouse control efficacy (60%) and field efficacy (30-56%)	[160]
Metarhizium brinneum	-	Reduce germination of microsclerotia, inhibited the hyphal formation	[256]
Paenibacillus polymyxa ShX301	Rhizosphere (Cotton)	Reduced disease incidence and disease severity	[257]
Pseudomonas fuorescences FS167	Root endophyte (Tomato)	Reduced mycelial growth, reduced disease index, chitinase, siderophores, protease	[249]
Pseudomonas mosselli FS67	Root endophyte (Tomato)	Reduced mycelial growth, reduced disease index, siderophores	[249]
Pseudomonas spp.	Rhizosphere (Olive)	Reduced disease onset and development, disease incidence, plant mortality, phytase, and catalase activities	[258]
Purpureocillium lilacinum QLP12	Soil (Eggplant)	Reduce disease index in greenhouse and field	[259]
Stenotrophomonas maltophilia FS300	Root endophyte (Tomato)	Reduced mycelial growth, reduced disease index, chitinase, siderophores, protease	[249]
Trichoderma atroviride		Antifungal activity	[154]

Biocontrol organism	Source	Mechanism of action /efficacy	References
Trichoderma harzianum	Rhizosphere (Pistachio)	Reduced mycelial growth and disease severity, mycoparasitism	[260]
Triterpene derivatives (at 10 µg/ml)	Latex (Euphorbia)	Reduced disease severity, stunting, and vascular discoloration; enhanced peroxidase and polyphenol oxidase activities in tomato	[158]

Table S3. Some examples of organic amendments (OAs) with suppressiveness against *Verticillium dahliae.*

Organic amendments	Test crop/source	Mechanism of action	References
Solid olive oil waste compost	Olive	Reduce mycelial growth; and microsclerotia viability (52%-76%)	[261]
Olive mill compost	Cotton	Low β-glucosidase activity; high oligotrophic actinomycete populations,<br>Mycelial growth inhibition,<br>Reduced disease severity, lower microsclerotial concentrations	[262]
Broccoli residue	Rotation with eggplant	Reduced disease incidence by 53%, reduced *V. dahliae* DNA in soil	[263]
Compost tea	Corn straw	Reduced mycelial growth and conidial germination by 91% and 78% respectively in strawberry; control efficacy of 42% in greenhouse	[264]

Organic amendments	Test crop/source	Mechanism of action	References
Grape marc compost	Olive	Reduced microsclerotia density; disease incidence;	[156,265]
Plant compost (grape pomace compost; olive pomace/dairy manure compost; mixed crop residue compost)	Bell pepper	reduced pathogen population in fields (29%-42%) up to 14 weeks post-application; Compost extracts reduced *V. dahliae* growth by 25-50% in Petri dish assay	[163]
Fresh manure (4 kg m^{-2} sheep manure + 1 kg m^{-2} composted poultry litter) covered with transparent plastic	Bell pepper	Reduced disease incidence to less than 1%	[167]
4% Dried spearmint or oregano	Tomato	Reduced disease to visually no symptoms	[266]
Composts with various compositions	Eggplant	Reduced disease indicated by disease suppression index	[267]
Tomato waste compost	Eggplant	Reduced disease severity and fungal colonization	[268]
Turkey litter compost	Eggplant	Reduced disease severity	[268]
Various compositions of steam-explosion liquid waste, Agro-industrial residues/waste, and plant green waste composts	Eggplant	Reduced disease indicated by disease suppression index (43%-65%), showed richness in potential biocontrol agents	[166]

Organic amendments	Test crop/source	Mechanism of action	References
Mustard	Tomato	Suppressed verticillium wilt with "biofumigant" properties	[164]
Sudangrass	Tomato	Suppressed verticillium wilt with "biofumigant" properties	[164]
Plant-based compost	Tomato	Reduced disease intensity, showed richness in culturable biocontrol agents	[162]
Compost of winery residues including grape stalks and grape pomace	Eggplant	Reduced disease incidence, severity and plant mortality, high total phenol content	[269]
Compost of tomato pulp with sawdust and chipping wood as bulking agents with a ratio of 2:1:1 v/v/v	Eggplant	Reduced disease incidence, severity and plant mortality, high total phenol content	[269]
Compost of organic fraction of municipal solid waste	Eggplant	Reduced disease incidence, severity and plant mortality, high total phenol content	[269]
Compost consisted of 73% olive mill extracted press cake, 24% wastewater, and 3% olive leaves	Eggplant	Reduced disease incidence, severity and plant mortality, high total phenol content	[269]

Table S4. Examples of sources of carbon from recent studies for anaerobic soil disinfestation of soil-borne pathogens including *Verticillium dahliae.*

The carbon source for ASD	Host plant	Targeted pathogen	References
Alfalfa	Cucumber	*Rhizoctonia solani*	[174,270]
Bean dregs	-	*Fusarium oxysporum*	[137]
Broccoli + Rice bran	Strawberry	*Fusarium oxysporum* f. sp. *fragariae; Verticillium dahliae*	[271]
Corn straw	-	-	[174]
Ethanol	Tomato; Cucumber; Strawberry	*Fusarium oxysporum* f. sp. *lycopersici; Fusarium oxysporum; Verticillium dahliae; Pyrenpchaeta lycopersici; Colletotrichum coccodes; Rhizoctonia solani Meloidogyne* spp.	[137,140,270,272,273]
FL-104 rye + Rice bran	Strawberry	*Fusarium oxysporum* f. sp. *fragariae*	[271]
Grape pomace	Strawberry	*Verticillium dahliae*	[140,175,274]
Italian rye + Rice bran	Strawberry	*Fusarium oxysporum* f. sp. *fragariae*	[271]
Molasses	Tomato Lettuce; Mustard green	*Plasmodiophora brassicae; Meloidogyne* spp.	[169,273,275]

The carbon source for ASD	Host plant	Targeted pathogen	References
		Verticillium dahliae; Pyrenpchaeta lycopersici; Colletotrichum coccodes	
Molasses + composted poultry litter (CPL)	Tomato	*Meloidogyne* spp.	[276]
Mustard + Rice bran	Strawberry	*Fusarium oxysporum* f. sp. *fragariae*	[271]
Mustard seed meal	Strawberry	*Verticillium dahliae*	[140,169]
Onion waste	Strawberry	*Verticillium dahliae*	[140]
Rice bran	Strawberry	*Verticillium dahliae; Fusarium oxysporum* f. sp. *fragariae*	[140,169,271]
Sudan grass + Rice bran	Strawberry	*Fusarium oxysporum* f. sp. *fragariae*	[271]
Sugarcane bagasse	-	*Fusarium oxysporum*	[137]

The carbon source for ASD	Host plant	Targeted pathogen	References
Tomato pomace	-	-	[169,175,274]
Triticale + rice bran	Strawberry	*Fusarium oxysporum* f. sp. *fragariae*	[271]
Wheat bran	Tomato; Strawberry	*Plasmodiophora brassicae*; Meloidogyne spp.<br>*Verticillium dahliae*; *Pyrenpchaeta lycopersici*; *Colletotrichum coccodes*; Meloidogyne spp.	[140,273,275]
Wheat bran + Molasses	Lettuce; Mustard green	*Plasmodiophora brassicae*; Meloidogyne spp.	[275]
